# Understanding the expression of loneliness on Twitter across age groups and genders

**DOI:** 10.1371/journal.pone.0273636

**Published:** 2022-09-28

**Authors:** Anietie Andy, Garrick Sherman, Sharath Chandra Guntuku

**Affiliations:** 1 Penn Medicine, University of Pennsylvania, Philadelphia, PA, United States of America; 2 Computer and Information Science, University of Pennsylvania, Philadelphia, PA, United States of America; Uniwersytet Jagiellonski w Krakowie Biblioteka Jagiellonska, POLAND

## Abstract

Some individuals seek support around loneliness on social media forums. In this work, we aim to determine differences in the use of language by users—in different age groups and genders (female, male), who publish posts on Twitter expressing loneliness. We hypothesize that these differences in the use of language will reflect how these users express themselves and some of their support needs. Interventions may vary depending on the age and gender of an individual, hence, in order to identify high-risk individuals who express loneliness on Twitter and provide appropriate interventions for these users, it is important to understand the variations in language use by users who belong to different age groups and genders and post about loneliness on Twitter. We discuss the findings from this work and how they can help guide the design of online loneliness interventions.

## 1 Introduction

Some individuals turn to social media and online forums to seek support and express themselves about their health and well-being. Prior work has used language features to gain insights from data collected from these social media and online forums as it relates to, e.g.: COVID-19 [1, 2], cancer [3–7], substance use recovery [8, 9], healthcare facilities [10], patient/provider experiences in healthcare [11, 12], and predicting risk of cardiovascular disease [13].

Loneliness is a public health concern; in the United States (US), it has been shown to have an effect on the mental and physical well-being of individuals [14–16]. Prior work which used data from social media and online forums to study loneliness focused on: (i) studying how users who seek support around loneliness by publishing posts on an online forum focused on discussions around loneliness (loneliness forum) communicate in other online forums not related to loneliness (non-loneliness forum) [17, 18] and (ii) understanding and characterizing individuals who express loneliness on Twitter and the responses they receive [15, 16, 19]. In [15], it was determined that posts by individuals who express loneliness on Twitter correlated with mental health predictors. [16] developed a categorization scheme for expressions of loneliness on social media posts. In [19], it was demonstrated that the online activity of users who express loneliness often on Twitter tend to be low.

The analyses in prior work did not take into consideration the differences in the way individuals of different age groups and genders communicate on social media platforms. In [20], it was determined that on social media, female users tend to express themselves differently when compared to male users. In [21], it was demonstrated that there are differences in language use by users in various age groups on social media.

Prior works used an open vocabulary approach based on the topic modeling algorithm, Latent Dirichlet Allocation (LDA) [22] and a psycho-linguistic dictionary, Linguistic Inquiry Word Count (LIWC) [23]—which consists of 73 categories such as personal pronouns and health and a list of curated words associated with each of these categories, to characterize and understand users who express [15, 17, 18], predict patients risk for cardiovascular disease [13], and to determine the types of social supports users seek on online health forums [1, 8]. Similarly, in this work, we identify users who express loneliness on Twitter and using LDA and LIWC, we do the following:

- We split these users into different age groups and analyze their tweets to determine the language use differences by users in these age groups who express loneliness on Twitter.
- We split these users based on their gender, i.e., female and male, and analyze the tweets by users belonging to each gender to determine the language use differences between female and male users who express loneliness on Twitter.
- We study the differences in the use of language among users belonging to different genders and age groups; for example, do female users who publish tweets expressing loneliness and belong to different age groups communicate differently?

We hypothesize that the LDA topics and LIWC categories extracted from tweets by users who belong to these different groups will reflect how these users express themselves, capture some of their support needs, and inform online loneliness interventions.

In this work, the effect sizes were measured using Cohen’s D, which indicates the standardized difference between two means and only results with Cohen’s D above or equal to 0.100 are reported. To indicate meaningful correlations, Benjamini-Hochberg p-correction was used with p<0.001 as our significance level.

## 2 Materials and methods

### 2.1 Ethics and privacy

This work was considered exempt based on the authors institutions Institutional Review Board guidelines. The data set used for the analysis in this paper are publicly available and for all our analysis, we de-identified the user names by using a unique number to represent each user.

### 2.2 Related work

Understanding how individuals express loneliness on social media platforms is important for identifying high-risk lonely individuals and for providing appropriate and relevant interventions to these individuals.

Using data from social media and online forums, several prior works predicted patients risk for health conditions and aimed to understand how people communicate as it relates to their health and well-being and the types of support they seek and give around health and well-being on these forums. In [13], data from electronic medical records and social media was used to predict patients risk for a cardiovascular disease. In [1, 2], data from an online forum focused on discussions around COVID-19 were analyzed to better understand the support needs of people who publish posts on these forums. In [3–7] data from online cancer forums were analyzed to categorize users and understand the support needs of members of these forums. In [24–26], social media data related to the mental health and well-being of users were analyzed.

As it relates to loneliness, prior works determined that loneliness is associated with depression [27] and has an effect on individual well-being [28]. Some prior work focused on using social media and online forum data to better understand the support needs of people who express the feeling of loneliness on these forums. For example, in [15], Twitter posts of users who expressed the feeling of loneliness were analyzed and it was determined that there was a correlation between posts by these users and mental health predictors. In [16], using data from a social media forum, a categorization scheme was developed for the expressions of loneliness and to determine their association with theories on loneliness. In [17], it was determined that users who self-declare to be depressed and express loneliness in posts published on an online loneliness forum communicate differently from users who do not declare to be depressed but express loneliness in posts published on an online loneliness forum. Using language features, [18] studied the differences in the way the same users communicate in an online loneliness forum compared to an online non-loneliness forum. In [29], it was determined that the language used to declare loneliness on social media varies by day and time. [30, 31] studied the expression of loneliness on social media during the COVID-19 pandemic. In [32] it was determined that the expression of loneliness in forums that are related to young adults varies from other forums and that coping strategies vary depending on the form of loneliness. [33] determined that there are differences in language use on social media around solitude compared to loneliness.

The analyses done in this work are different from those from prior work; in this work, we aim to show the differences in language use in Twitter posts by users—belonging to different age groups and genders, that publish Twitter posts expressing loneliness.

### 2.3 Data set

We use the data set from [15], which contains 408,296,620 tweets and metadata about the authors of these tweets. Using this data set, prior work [15] determined that users who published tweets in which they mentioned the words *“alone”* or *“lonely”* 5 or more times tended to express their feeling of loneliness compared to users with fewer mentions of *“alone”* or *“lonely”* in their tweets. From this data set [15], we identified users with 5 or more tweets mentioning either *“alone”* or *“lonely”* and that had published at least 50 tweets; for each of these users, we collected the user id’s and each user’s most recent 3,200 tweets, published between 2012 and 2016. For the analysis in this work, the tweets that mentioned either *“alone”* or *“lonely”* were removed to reduce the likelihood of identifying the users who expressed loneliness. We end up with 14,847,123 tweets by 6,202 users who had 5 or more published tweets in which they mentioned either *“alone”* or *“lonely”*.

Using social media data, prior work [21] studied the differences in language use by users in the following age groups: *13–18*, *19–22*, *23–29*, and *30–65*. Similarly, for the analysis in this work, we focus on using social media data from users belonging to the following age groups: *18–22*,*23–29*, and *30–65*. On social media forums, users rarely self-declare their age and gender. For example, to identify how many users in our data set self-declared their age, we selected tweets that mentioned either *“i am”* or *“i’m”* followed by a number and the phrase *“years old”’*; we identified 27 users who self-declared their age. Similarly, to identify users who self-declared their gender as female in our data set, we selected tweets that mentioned either *“i am”* or *“i’m”* followed by either *“female”*, *“a female”*, *“a girl”* or *“a woman”* and observed that 109 users self-declared to be female. We used the same approach to identify users who self-declared to be male, using *“male”*, *“a male”*, *“a man”*, *“a boy”*, or *“a guy”* in place of the female phrases; we observed that 90 users self-declared to be male.

Using social media data and a gender and age predictive lexica, prior work [34] predicted the gender and age of social media users with high accuracy and it was shown to generalize to other data sets; the predictive lexica from [34] has been used to determine the gender and age of social media users in several prior works [35–37]. Hence in this work, we use the gender and age predictive lexica from [34] to estimate the gender (female and male) and age of users in our data set. We tested this gender and age predictive lexica [34] on tweets by users in our data set who self-declared their age and gender and observed that: (a) for age, the predictive lexica accurately predicted the age of 19 out of 27 users and for the other users it approximated their age to within 1–2 years of their actual age. Also, we observed that for all the users in which the predictive lexica approximated their ages, the predicted age for each of these users fell within the age range (i.e. *18–22*, *23–29*, and *30–65*) in which the actual age of these users belonged to; for example, if a users actual age was 19, the predicted age was between 18 and 22 (b) For gender, out of the 109 users who self-declared to be female, the predictive lexica accurately predicted 101 and out of the 90 users who self-declared to be male, the predictive lexica accurately predicted 83.

A potential control group of users was identified by matching each user (with at least 5 tweets mentioning *“alone”* or *“lonely”*) to another user (who had never mentioned either *“alone”* or *“lonely”* in a tweet) by period of activity, i.e., the dates of the first and last tweets, and by age and gender; the age and gender of the control group users were also determined using [34]. In our analysis, we excluded non-English tweets and retweets.

Similar to prior work [15], using LDA as implemented by MALLET [38], we generated 200 topics using tweets from users in our data set and the control group.

This paper is formatted as follows: using LDA and LIWC: (a) in section **Age group analysis**, we identify the LDA topics and LIWC categories associated with users who belong to different age groups and have 5 or more tweets that mention either *“alone”* or *“lonely”*, (b) in section **Gender analysis**, we identify the LDA topics and LIWC categories associated with users who belong to different genders and have 5 or more tweets that mention either *“alone”* or *“lonely”*, and (c) in section **gender and age group analysis**, we identify the LDA topics and LIWC categories associated with users who belong to different genders and age groups.

In this work, we also make reference to users who have 5 or more tweets that mention either *“alone”* or *“lonely”* as users who express loneliness. For all the analysis in each section in this work, the control group users were matched by age, gender, and period of activity i.e. the dates of their first and last Twitter posts.

### 2.4 Age group analysis

In this section, we use LDA and LIWC to show the differences in the use of language by users who publish tweets expressing loneliness and belong to the following age groups: *18–22*, *23–29*, and *30–65* compared to their corresponding control group users. From our data set, we identify users who belong to these different age groups and match them with their corresponding control group users; below, we describe the data set used for the analysis in this section:

- For users between ages 18 and 22, we identified 4,203 users who expressed loneliness; these users published 6,865,033 tweets. We matched each user expressing loneliness with a control group user; these control group users published 3,342,625 tweets
- For users between ages 23 and 29, we identified 1,631 users who expressed loneliness; these users published 2,636,371 tweets. Each user was matched with a control group user; the control group users published 1,246,166 tweets
- For users between ages 30 and 65, we identified 175 users who expressed loneliness and these users published 266,115 tweets. We matched each of these users with a control group user; these control group users published 98,746 tweets

In order to determine the LDA topics that are most associated with Twitter posts belonging to each of the target age groups that express loneliness on Twitter compared to their corresponding control groups, we did the following: (i) we identify single words in Twitter posts by users belonging to these different groups by using the HappierFunTokenizer tokenization tool: (https://github.com/dlatk/happierfuntokenizing/)—which is a tokenization tool that can identify words, variations of word spellings, and emoticons (ii) we then used the MALLET [38, 39] implementation of the LDA algorithm. LDA, which groups words that co-occur in documents (i.e. Twitter posts in this work), is a generative model which makes the following assumptions: (a) topics are made of combinations of tokens or words and (b) Twitter posts are made up of topic combinations; Gibbs sampling [40] may be used for estimating the latent variables associated with the topics since the words associated with the Twitter posts are known. A label can be associated with each topic based on the context words associated with the topics e.g. the LDA algorithm may group the following words together *“winter”*, *“spring”*, *“summer”*, *“fall”* as seasons of the year.

Using the 200 LDA topics generated from our data set (described in section **Data Set**) and the data sets described in this section, similar to prior works which used LDA as implemented by MALLET [38] to: (a) compare social media posts by users belonging to different age groups to identify the LDA topics themes most associated with users belonging to these groups [21] and (b) compare posts by users who express loneliness on social media and online forums to posts by users who do not express loneliness on social media/online forums and identified the topic themes most associated with users in each of these groups [15, 18]; in this work we use the MALLET [38] implementation of LDA to compare and identify the topic themes most associated with the Twitter posts belonging to each of the target age groups that express loneliness on Twitter compared to tweets by their corresponding control group users.

For all the posts belonging to each of the target age groups that express loneliness on Twitter, similar to prior work which identified the proportion of LIWC categories most correlated with social media posts by: (a) users who express loneliness on online forums and Twitter compared to those who do not [15, 18] and (b) users who are at risk for cardiovascular disease compared to those who are not [13]; in this work, we compare the proportion of LIWC categories correlated with words in posts published on Twitter by users who express loneliness on Twitter and belong to each of the target age groups compared to tweets by their corresponding control group users.

### 2.5 Age group analysis: Results

#### 2.5.1 LDA analysis results

Tables 1–3 show the effect sizes (Cohen’s D) between the most significant LDA topic distributions of the users belonging to the three age groups with tweets expressing loneliness compared to their corresponding control users.

**Table 1:** Results from LDA analysis for users who express loneliness on Twitter and are between the ages of 18 and 22.

Topic No.	LDA Topics and Highly Correlated Words	Cohen’s D
1	hate, people, stupid, reason, annoying,fact piss, sumb, passion, haters	0.180
2	smoke, weed, roll, blunt, high, drug, drink, dope, blow, cigarettes	0.179
3	sleep, awake, tired, bed, wide, wake, nap, goodnight, sleepy, exhausted	0.177
4	bad, sick, stomach, hurt, body, headache, pain, throat, cough, sore	0.171
5	food, eat, pizza, breakfast, hungry, dinner, cook, ate, order, meal	0.148
6	hurt, feelings, trust, forget, care, truth, person, lie, matter, lose	0.147

**Table 2:** Results from LDA analysis for users who express loneliness on Twitter and are between the ages of 23 and 29.

Topic No.	LDA Topics and Highly Correlated Words	Cohen’s D
1	hate, people, stupid, reason, annoying, absolutely, fact, dumb, passion, haters	0.182
2	relationship, single, person, matter, boyfriend, perfect, girlfriend, fight, loyal, worth	0.169
3	sleep, awake, tired, bed, wide, woke, nap, goodnight, sleepy, exhausted	0.167
4	food, eat, pizza, breakfast, hungry, dinner, cook, ate, order, meal	0.161
5	bad, sick, stomach, hurt, head, killing, pain, throat, cough, sore	0.160
6	smoke, weed, roll, blunt, high, drug, drink, dope, blow, cigarettes	0.156

**Table 3:** Results from LDA analysis for users who express loneliness on Twitter and are between the ages of 30 and 65.

Topic No.	LDA Topics and Highly Correlated Words	Cohen’s D
1	music, listen, album, song, video, track, beats, download, sounds, single	0.184
2	smoke, weed, roll, blunt, high, drug, drink, dope, blow, cigarettes	0.178
3	mom, dad, told, sister, brother, called, grandma, boyfriend, aunt, daughter	0.159
4	bad, sick, stomach, hurt, head, killing, pain, throat, cough, sore	0.133
5	people, talk, understand, judge, act, rude, treat, opinion, respect, complain	0.130
6	man, woman, treat, king, queen, respect, beautiful, grown, lady, strong	0.125
7	dog, cat, puppy, cute, animal, pet, kitty, kitten, meow, puppies	0.100

We observed that users between the ages of 18 and 22 and those between 23 and 29 tended to publish posts on topic themes related to sleep, food, and substance use. However users between 18 and 22 tended to publish posts on topic themes about trust and their feeling being hurt by others (topic 6 in Table 1) compared to users between 23 and 29 who tended to post more about topics on fighting, loyalty, and worth in relationships (topic 2 in Table 2). Users between ages 30 and 65 tend to post about topics related to listening to music, communicating with family members, how people treat/view/talk about others, expressing compliments and how one should be treated with respect, and post about pets, as shown by topics 1, 3, 5, 6, and 7, respectively in Table 3.

#### 2.5.2 LIWC analysis results

Below, we show the effect sizes (Cohen’s D) and the LIWC categories most associated with users belonging to the three age groups compared with their corresponding control group users.

The following LIWC categories (and corresponding effect sizes i.e. Cohen’s D) were associated with users belonging to the age group 18—22: *First person singular pronoun* (Cohen’s D = 0.201), *Negations* (Cohen’s D = 0.178), *Negative emotion* (Cohen’s D = 0.153), *Focus present* (Cohen’s D = 0.131), and *Anger* (Cohen’s D = 0.129).

The following LIWC categories (and corresponding effect sizes i.e. Cohen’s D) were associated with users belonging to the age group 23—29: *First person singular pronoun* (Cohen’s D = 0.184), *Negations* (Cohen’s D = 0.170), *Negative emotion* (Cohen’s D = 0.159), *Anger* (Cohen’s D = 0.135), and *Anxiety* (Cohen’s D = 0.100).

The following LIWC categories (and corresponding effect sizes i.e. Cohen’s D) were associated with users belonging to the age group 30—65: *First person singular pronoun* (Cohen’s D = 0.153), *Hear* (Cohen’s D = 0.135), *Negative emotion* (Cohen’s D = 0.114), and *Sadness* (Cohen’s D = 0.100).

We observed that the LIWC categories on *Anxiety* and *Sadness* were more associated with users between ages 23—29 and 30—65, respectively. We discuss these results in the discussion section.

### 2.6 Gender analysis

In this section, we use LDA and LIWC to show the differences in the use of language by female and male users who publish posts expressing loneliness on Twitter compared to a control group of female and male users, respectively. From our data set, (described in section **Data Set**), we identified users belonging to different genders (female and male) and matched them with their corresponding control group users; we describe the data set used for the analysis in this section below.

- We identified 4,400 female users who expressed loneliness; these users published 7,085,207 tweets. Each user expressing loneliness was matched with a control user; the control users published a total of 3,295,941 tweets
- We identified 1,802 male users who expressed loneliness; these users published 3,004,436 tweets. We matched each user with a control group user; these control group users published 1,461,539 tweets

Similar to section **Age group analysis**, we use the 200 LDA topics generated from our data set to identify the LDA topic themes which are most associated with tweets published by female and male users, respectively, with 5 or more tweets that include the words *“alone”* or *“lonely”* compared to their corresponding matched control users. Also, similar to section **Age group analysis**, we identify the LIWC categories most associated with Twitter posts by users who express loneliness on Twitter and belong to different genders (female and male) compared to tweets by their corresponding control group users.

### 2.7 Gender analysis: Results

#### 2.7.1 LDA analysis results

Tables 4 and 5 show the effect sizes (Cohen’s D) between the most significant LDA topic distributions of the female and male users, respectively, with tweets expressing loneliness compared to their corresponding control users. We observed that female users tend to express their feelings/emotions such as happiness/sadness/anger and feeling scared/mad/confused/upset/afraid/jealous, as shown in topics 8 and 9, respectively in Table 4 compared to male users who tend to post more topics related to hate/annoyance with people, feeling sick, and issues with trust and problems in relationships, as shown in topics 2, 5, and 9, respectively in Table 5.

**Table 4:** Results from LDA analysis for gender: Female.

Topic No.	LDA Topics and Highly Correlated Words	Cohen’s D
1	sleep, awake, tired, bed, wide, wake, nap, goodnight, sleepy, exhausted	0.251
2	smoke, weed, roll, blunt, high, drug, drink, dope, blow, cigarettes	0.228
3	music, listen, album, song, video, track, beats, download, sounds, single	0.199
4	relationship, single, person, matter, boyfriend, perfect, girlfriend, fight, loyal, worth	0.198
5	hurt, feelings, trust, forget, care, truth, person, lie, matter, lose	0.195
6	love, fall, asleep, fell, falling, boy, falls, place, arms, inlove	0.156
7	hold, hand, kiss, touch, lips, fingers, hug, arms, tight, kisses	0.133
8	make, feel, happy, sense, laugh, sad, cry, smile, special, angry	0.123
9	scared, mad, confused, upset, obsessed, sad, annoyed, afraid, jealous, proud	0.107

**Table 5:** Results from LDA analysis for gender: Male.

Topic No.	LDA Topics and Highly Correlated Words	Cohen’s D
1	relationship, single, person, matter, boyfriend, perfect, girlfriend, fight, loyal, worth	0.331
2	hate, people, stupid, reason, annoying, absolutely, fact, dumb, passion, haters	0.303
3	smoke, weed, roll, blunt, high, drug, drink, dope, blow, cigarette	0.296
4	hurt, feelings, trust, forget, care, truth, person, lie, matter, lose	0.284
5	bad, sick, stomach, hurt, body, headache, pain, throat, cough, sore	0.281
6	sleep, awake, tired, bed, wide, woke, nap, goodnight, sleepy, exhausted	0.256
7	love, fall, asleep, fell, falling, boy, falls, place, arms, inlove	0.245
8	fuck, give, gave, chance, advice, deserve, credit, attention, attitude, hug	0.231
9	problem, relationship, trust, lack, respect, loyalty, fear, attitude, attention, happiness	0.221
10	hold, hand, kiss, touch, lips, fingers, hug, arms, tight, kisses	0.218

#### 2.7.2 LIWC analysis results

Below are the effect sizes (Cohen’s D) and the LIWC categories most associated with female and male users when compared to their corresponding control group users.

The following LIWC categories (with their effect sizes i.e. Cohen’s D) were associated with female users: *First person singular pronoun* (Cohen’s D = 0.255), *Negations* (Cohen’s D = 0.247), *Negative emotion* (Cohen’s D = 0.222), *Anger* (Cohen’s D = 0.184), *Focus present* (Cohen’s D = 0.179), *Risk* (Cohen’s D = 0.140), *Sadness* (Cohen’s D = 0.132), *Focus future* (Cohen’s D = 0.119), *Health* (Cohen’s D = 0.116).

Similarly, the following LIWC categories (with their effect sizes i.e. Cohen’s D) were associated with male users: *First person singular pronoun* (Cohen’s D = 0.336), *Female references* (Cohen’s D = 0.286), *Negations* (Cohen’s D = 0.252), *Negative emotion* (Cohen’s D = 0.212), *Anger* (Cohen’s D = 0.200), *Focus present* (Cohen’s D = 0.160), *Health* (Cohen’s D = 0.130), *Anxiety* (Cohen’s D = 0.116).

We observe that the LIWC categories on *Sadness*, *Risk*, and *Focus future* were more associated with female users and the LIWC categories on *Female references* and *Anxiety* were more associated with male users.

We discuss these findings in the discussion section.

### 2.8 Gender and age group analysis

In this section, we use LDA and LIWC to show the differences in the use of language by users who belong to different age groups and genders and express loneliness on Twitter. From our data set (section **Data set**), we identified female users who expressed loneliness on Twitter and grouped them into the target age groups: *18—22*, *23—29*, and *30—65*; we then matched these female users in these groups with their corresponding control group users. Similarly, from our data set, we identified male users who expressed loneliness on Twitter and grouped them into the following groups: *18—22*, *23—29*, and *30—65*; these male users in each of these groups where then matched with their corresponding control users. Below we describe the data set used for the analysis in this section:

- We identified 3,099 female users who expressed loneliness on Twitter and belonged to the age group *18—22*; we collected 5,003,956 tweets published by these users. We matched each of these users with corresponding control group users; the control group users published 2,421,620 tweets
- We identified 1,023 female users (with 1,635,254 published tweets) who published tweets expressing loneliness and belonged to the age group *23—29*. We matched each of these users with their corresponding control group users who published 750,167 tweets
- We identified 108 female users (with 162,364 tweets) who published tweets expressing loneliness and belonged to the age group *30—65* and we matched each of these users with corresponding control group users; these control group users published 61,403 tweets
- We identified 1,104 male users (with 1,861,077 published tweets) who published tweets expressing loneliness and belonged to the age group *18—22*. We matched each user with a corresponding control group user; these control group users published 921,005 tweets
- We identified 608 male users (with 1,001,117 published tweets) who published tweets expressing loneliness and belonged to the age group *23—29*. We matched each user with a corresponding control group user; these control group users published 495,999 tweets
- We identified 67 male users (with 103,751 published tweets) who published tweets expressing loneliness and belonged to the age group *30—65*. We matched each of these users with a corresponding control group user; these control group users published 37,343 tweets.

Using the 200 LDA topics generated from our data set (**section Data set**) and the data set described in this section and LIWC, we identify the topic themes and LIWC categories most associated with tweets by users belonging to each of these groups compared to their corresponding control groups.

### 2.9 Gender and age group analysis: Results

Here, we report the results from two sets of analyses, i.e. (a) the differences in topic themes and LIWC categories associated with users who belong to the same gender but different age groups and (b) the differences in topic themes and LIWC categories most associated with users who belong to different genders but the same age group.

#### 2.9.1 Users who belong to the same gender but different age groups

Below we report the results from the analysis in this section.

Tables 6–8 show the LDA topics most associated with male users who express loneliness and belong to the age groups *18—22*, *23—29*, and *30—65*, respectively. Also, Tables 9–11 show the LDA topics associated with female users who express loneliness and belong to these same age groups.

**Table 6:** Results from LDA analysis for male: 18—22.

Topic No.	LDA Topics and Highly Correlated Words	Cohen’s D
1	relationship, single, person, matter, boyfriend, perfect, girlfriend, fight, loyal, worth	0.737
2	smoke, weed, roll, blunt, high, drug, drink, dope, blow, cigarettes	0.707
3	hate, people, stupid, reason, annoying, absolutely, fact, dumb, passion, haters	0.692
4	hurt, feelings, trust, forget, care, truth, person, lie, matter, lose	0.683
5	bad, sick, stomach, hurt, head, killing, pain, throat, cough, sore	0.662
6	love, fall, asleep, fell, falling, boy, falls, place, arms, inlove	0.554
7	music, listen, album, song, video, track, beats, download, sounds, single	0.520
8	problem, relationship, trust, lack, respect, loyalty, fear, attitude, attention, happiness	0.471
9	hold, hand, kiss, touch, lips, fingers, hug, arms, tight, kisses	0.446
10	make, feel, happy, sense, laugh, sad, cry, smile, special, angry	0.431
11	friends, family, close, true, fake, thankful, zone, hang, enemies, lucky	0.399
12	girls, guys, boys, men, women, attractive, female, dudes, ugly, male	0.346
13	tired, bored, sick, hungry, hell, sleepy, annoyed, irritated, soo, lazy	0.279
14	scared, mad, confused, upset, obsessed, sad, annoyed, afraid, jealous, proud	0.252
15	drunk, tonight, bar, shots, fun, sober, beer, alcohol, 21, faded	0.227
16	pain, stress, anxiety, brain, mental, death, depression, anger, panic, health	0.225

**Table 7:** Results from LDA analysis for male: 23—29.

Topic No.	LDA Topics and Highly Correlated Words	Cohen’s D
1	relationship, single, person, matter, boyfriend, perfect, girlfriend, fight, loyal, worth	0.775
2	hate, people, stupid, reason, annoying, fact piss, sumb, passion, haters	0.645
3	smoke, weed, roll, blunt, high, drug, drink, dope, blow, cigarettes	0.571
4	love, fall, asleep, fell, falling, boy, falls, place, arms, inlove	0.548
5	bad, sick, stomach, hurt, body, headache, pain, throat, cough, sore	0.534
6	problem, relationship, trust, lack, respect, loyalty, fear, attitude, attention, happiness	0.507
7	fuck, give, gave, chance, advice, deserve, credit, attention, attitude, hug	0.502
8	hurt, feelings, trust, forget, care, truth, person, lie, matter, lose	0.495
9	sleep, awake, tired, bed, wide, wake, nap, goodnight, sleepy, exhausted	0.492
10	girl, guy, boy, she’s, cute, he’s, boyfriend, girlfriend, likes, date	0.483
11	man, woman, treat, king, queen, respect, beautiful, grown, lady, strong	0.469
12	music, listen, album, song, video, track, beats, download, sounds, single	0.417
13	friends, family, close, true, fake, thankful, zone, hang, enemies, lucky	0.361
14	time, waste, spend, remember, wasted, worth, nap, quality, spent, energy	0.347
15	girls, guys, boys, men, women, attractive, female, dudes, ugly, male	0.339
16	drunk, tonight, bar, shots, fun, sober, beer, alcohol, 21, faded	0.326
17	mom, dad, told, sister, brother, called, grandma, boyfriend, aunt, daughter	0.303
18	love, fall, asleep, fell, falling, boy, falls, place, arms, inlove	0.283
19	eyes, open, tears, close, cry, blind, mouth, joy, pain, smile	0.271
20	make, feel, happy, sense, laugh, sad, cry, smile, special, angry	0.231
21	tired, bored, sick, hungry, hell, sleepy, annoyed, irritated, soo, lazy	0.194

**Table 8:** Results from LDA analysis for male: 30—65.

Topic No.	LDA Topics and Highly Correlated Words	Cohen’s D
1	music, listen, album, song, video, track, beats, download, sounds, single	0.857
2	bad, sick, stomach, hurt, body, headache, pain, throat, cough, sore	0.743
3	smoke, weed, roll, blunt, high, drug, drink, dope, blow, cigarettes	0.701
4	video, added, playlist, music, drinking, official, youtube, lyrics, minecraft, uploaded	0.665
5	music, listening, country, pandora, song, radio, station, blasting, loud, rap	0.656
6	kids, child, parents, married, young, son, wife, father, mother, daughter	0.645

**Table 9:** Results from LDA analysis for female: 18—22.

Topic No.	LDA Topics and Highly Correlated Words	Cohen’s D
1	sleep, awake, tired, bed, wide, wake, nap, goodnight, sleepy, exhausted	0.524
2	hate, people, stupid, reason, annoying, absolutely, fact, dumb, passion, haters	0.513
3	smoke, weed, roll, blunt, high, drug, drink, dope, blow, cigarettes	0.508
4	bad, sick, stomach, hurt, body, headache, pain, throat, cough, sore	0.501
5	music, listen, album, song, video, track, beats, download, sounds, single	0.433
6	relationship, single, person, matter, boyfriend, perfect, girlfriend, fight, loyal, worth	0.417
7	hurt, feelings, trust, forget, care, truth, person, lie, matter, lose	0.390
8	love, fall, asleep, fell, falling, boy, falls, place, arms, inlove	0.317
9	video, added, playlist, music, drinking, official, youtube, lyrics, minecraft, uploaded	0.289
10	hold, hand, kiss, touch, lips, fingers, hug, arms, tight, kisses	0.279
11	time, waste, spend, remember, wasted, worth, nap, quality, spent, energy	0.264
12	fuck, give, gave, chance, advice, deserve, credit, attention, attitude, hug	0.261
13	problem, relationship, trust, lack, respect, loyalty, fear, attitude, attention, happiness	0.261
14	pain, stress, anxiety, brain, mental, death, depression, anger, panic, health	0.255
15	make, feel, happy, sense, laugh, sad, cry, smile, special, angry	0.254
16	scared, mad, confused, upset, obsessed, sad, annoyed, afraid, jealous, proud	0.243
17	game, win, team, lebron, heat, fan, bowl, eagles, lose, nba	0.229

**Table 10:** Results from LDA analysis for female: 23—29.

Topic No.	LDA Topics and Highly Correlated Words	Cohen’s D
1	sleep, awake, tired, bed, wide, wake, nap, goodnight, sleepy, exhausted	0.534
2	hate, people, stupid, reason, annoying, fact piss, sumb, passion, haters	0.519
3	bad, sick, stomach, hurt, body, headache, pain, throat, cough, sore	0.499
4	smoke, weed, roll, blunt, high, drug, drink, dope, blow, cigarettes	0.441
5	time, waste, spend, remember, wasted, worth, nap, quality, spent, energy	0.439
6	relationship, single, person, matter, boyfriend, perfect, girlfriend, fight, loyal, worth	0.433
7	fuck, give, gave, chance, advice, deserve, credit, attention, attitude, hug	0.401
8	hurt, feelings, trust, forget, care, truth, person, lie, matter, lose	0.398
9	music, listen, album, song, video, track, beats, download, sounds, single	0.379
10	love, fall, asleep, fell, falling, boy, falls, place, arms, inlove	0.319
11	problem, relationship, trust, lack, respect, loyalty, fear, attitude, attention, happiness	0.308
12	girl, guy, boy, she’s, cute, he’s, boyfriend, girlfriend, likes, date	0.303
13	shit, funny, mad, tho, af, lmao, ugly, hype, dumb, weak	0.270
14	make, feel, happy, sense, laugh, sad, cry, smile, special, angry	0.252
15	you’re, talking, mine, lucky, missing, jealous, asshole, idiot, lying, kidding	0.222
16	pain, stress, anxiety, brain, mental, death, depression, anger, panic, health	0.211

**Table 11:** Results from LDA analysis for female: 30—65.

Topic No.	LDA Topics and Highly Correlated Words	Cohen’s D
1	hurt, feelings, trust, forget, care, truth, person, lie, matter, lose	0.571
2	mom, dad, told, sister, brother, called, grandma, boyfriend, aunt, daughter	0.561
3	people, talk, understand, judge, act rude, treat, opinion, respect, complain	0.533
4	life, past, future, learn, move, forward, lesson, forget, plan, memories	0.518
5	love, fall, asleep, fell, falling, boy, falls, place, arms, inlove	0.515
6	problem, relationship, trust, lack, respect, loyalty, fear, attitude, attention, happiness	0.481
7	song, favorite, band, album, music, listen, lyrics, hear, guitar, theme	0.470
8	smoke, weed, roll, blunt, high, drug, drink, dope, blow, cigarettes	0.450
9	sleep, awake, tired, bed, wide, woke, nap, goodnight, sleepy, exhausted	0.433
10	man, woman, treat, king, queen, respect, beautiful, grown, lady, strong	0.405
11	made, decision, mistake, plans, choose, moves, regrets, choices, happen, easy	0.398

The following LIWC categories (and their Cohen’s D) were most associated with male users belonging to the age group *18–22*: *First person singular pronoun* (Cohen’s D = 0.386), *Negations* (Cohen’s D = 0.303), *Female references* (Cohen’s D = 0.280), *Negative emotion* (Cohen’s D = 0.240), *Focus present* (Cohen’s D = 0.212), *Anger* (Cohen’s D = 0.208), *Swear* (Cohen’s D = 0.206), *Third person plural* (Cohen’s D = 0.157), *Health* (Cohen’s D = 0.145), *Sadness* (Cohen’s D = 0.135), *Second person pronoun* (Cohen’s D = 0.111).

The following LIWC categories (and their Cohen’s D) were most associated with male users belonging to the age group *23–29*: *First person singular pronoun* (Cohen’s D = 0.338), *Female references* (Cohen’s D = 0.325), *Negations* (Cohen’s D = 0.240), *Swear* (Cohen’s D = 0.231), *Negative emotion* (Cohen’s D = 0.217), *Anger* (Cohen’s D = 0.204), *Social processes* (Cohen’s D = 0.195), *Anxiety* (Cohen’s D = 0.150), *Health* (Cohen’s D = 0.135), *Focus present* (Cohen’s D = 0.131).

The following LIWC categories (and their Cohen’s D) were most associated with male users belonging to the age group *30–65*: *First person singular pronoun* (Cohen’s D = 0.342), *Leisure* (Cohen’s D = 0.271).

The following LIWC categories (and their Cohen’s D) were most associated with female users belonging to the age group *18–22*: *First person singular pronoun* (Cohen’s D = 0.285), *Negations* (Cohen’s D = 0.256), *Negative emotion* (Cohen’s D = 0.228), *Feel* (Cohen’s D = 0.213), *Anger* (Cohen’s D = 0.195), *Focus present* (Cohen’s D = 0.192), *Risk* (Cohen’s D = 0.166), *Health* (Cohen’s D = 0.140).

The following LIWC categories (and their Cohen’s D) were most associated with female users belonging to the age group *23–29*: *Negations* (Cohen’s D = 0.266), *First person singular pronoun* (Cohen’s D = 0.261), *Negative emotion* (Cohen’s D = 0.253), *Anger* (Cohen’s D = 0.206), *Feel* (Cohen’s D = 0.201), *Focus present* (Cohen’s D = 0.155), *Anxiety* (Cohen’s D = 0.142), *Sadness* (Cohen’s D = 0.128), *Focus future* (Cohen’s D = 0.118).

The following LIWC categories (and their Cohen’s D) were most associated with female users belonging to the age group *30–65*: *Feel* (Cohen’s D = 0.250), *Negations* (Cohen’s D = 0.233), *Sadness* (Cohen’s D = 0.217), *Hear* (Cohen’s D = 0.216), *Negative emotion* (Cohen’s D = 0.208), *First person singular pronouns* (Cohen’s D = 0.173).

We observed that male users between the ages of 18 and 22 tend to post on topic themes related to intimacy in relationships, express more emotions (such as feeling scared, confused, afraid, and jealous), and post about mental health concerns, as shown in topics 9, 14, and 16, respectively in Table 6. Male users between ages 23 and 29 tend to post on topics related to dating / a boy or girl being cute, expressing compliments and how one should be treated with respect, as shown in topics 10 and topic 11 in Table 7. Male users between the ages of 30 and 65 tend to post about topics related to their family as shown in topic 6 in Table 8.

We observed that male users between the ages of 18 and 22 tended to use words from the LIWC categories on *sadness*, *third person plural*, and *second person pronoun*, while male users between the ages of 23 and 29 tended to use words from the LIWC categories associated with *anxiety* and *social processes*. Male Users between 30 and 65 tended to use more words from the LIWC category on *leisure*.

Regarding female users, we observed that female users between 18 and 22 tend to post about intimacy in relationships, express their feelings/emotions such as feeling scared/confused/afraid/jealous and tend to talk more about sports, as shown in topics 10, 16, and 17, respectively, in Table 9. Female users between 23 and 29 tend to discuss topics related to dating / a boy or girl being cute, as shown in topic 12 in Table 10. Female users between 30 and 65 tend to post on topics related to communicating with their family members, how people treat/view/talk about others, and their mistakes and regrets as shown in topics 2, 3, and 11 in Table 11.

We observed that female users between the ages of 18 and 22 tended to use more words from the LIWC categories on *risk* and *health*, while female users between the ages of 23 and 29 tended to use more words from the LIWC category on *anxiety* and *focusing on the future*. Female users between the ages of 30 and 65 tended to use more words from the LIWC category *Hear*.

#### 2.9.2 Users who belong to different genders but the same age group

We compare the LDA topics themes most associated with female and male users between the age group *18—22* who express loneliness and observed that: female users in this age group tended to post more on topic themes on insomnia and sports, respectively, as shown in topics 1 and 17, in Table 9 compared to male users who tend to post on topics about fake friends/family, about women and men being attractive/ugly, express being bored/irritated/tired/sleepy, and getting drunk, as shown in topics 11, 12, 13, and 15, respectively, on Table 6. Also, we observed that female users between 18 and 22 tended to use more words from the LIWC categories on *risk* and *feel* compared to male users between 18 and 22 who tended to use more words from the LIWC categories on *sadness* and *female references*.

Female users between 23 and 29 who express loneliness in our data set tended to post on topics themes related to mental health concerns as shown in topic 16 in Table 10 compared to male users between 23 and 29 who tend to post on topic themes related to expressing compliments and how one should be treated with respect, fake friends/family, men or women being attractive/ugly, getting drunk, communicating with family members, and feeling bored/irritated/tired/sleepy, as shown in topics 11, 13, 15, 16, 17, and 21, respectively in Table 7. Also, female users between 23 and 29 tend to use more words from the LIWC categories on *sadness*, *feel*, and *focus future* compared to male users between 23 and 29 who tended to use words from the LIWC categories on *female references*, *swearing*, *social processes*, and *health*.

Female users between the ages of 30 and 65 tended to post on topic themes on trust and their feelings being hurt, communicating with family members, how people treat/view/talk about others, companionship, problems such as trust and respect in relationships, issues with insomnia, compliments and how one should be treated with respect, and their mistakes and regrets, as shown in topics 1, 2, 3, 5, 6, 9, 10, and 11, respectively, in Table 11. Male users between the ages of 30 and 65 tend to post on topics related to feeling sick and family members as it relates to marriage, as shown in topics 2 and 6 in Table 8. Also, female users between 30 and 65 tended to use more words from the LIWC categories on *feelings*, *negations*, *sadness*, *hear*, and *negative emotion* compared to male users between 30 and 65 who tended to use more words from the LIWC category on *leisure*.

We discuss these results in the discussion section.

## 3 Discussion

In this work, using language features we determine the language use differences (which reflect some of the variations in support needs/concerns) expressed in Twitter posts by users who post about loneliness on Twitter and: (a) belong to different age groups and (b) are either female or male. In this section, we discuss our findings.

### 3.1 Age group analysis

As it relates to the age group analysis, we find that users in the age groups: *18—22* and *23—29* tend to publish Twitter posts about issues with sleep and substance use (Tables 1 and 2, respectively). The finding around users in this age group posting about substance use aligns with prior work [21], which showed that users between ages 19 to 22 and 23 to 29, respectively tend to talk more about substance use on social media. Prior work [15] determined that users who express loneliness on Twitter tended to post about topic themes on issues with sleep, however, in this work, we determine that users who express loneliness on Twitter and belong to the age groups *18—22* and *23—29* tend to express problems with sleep compared to users who belong to the age group *30—65*. Other findings show that users who express loneliness on Twitter and are between the ages of 18 and 22 tend to post more about topics related to trust and being hurt by others (Table 1), users between the ages of 23 and 29 tend to post more about topics related to fighting and loyalty in relationships (Table 2), and users between 30 and 65 tend to post more on topics related to communicating with their family members, how people treat/view/talk about others, and about their pets (Table 3). These findings indicate that while there are similarities in topics associated with users in different age group that express loneliness on Twitter, there are also differences; hence, when designing online loneliness interventions, it is important to take into consideration the age of the individuals. For example, given that users between the ages of 30 and 65 tend to post on topics themes related to their pets, potentially, this may indicate that these users like spending time with their pets; hence, an online loneliness intervention for users in this age group can provide suggestions and recommendations for getting a pet, if they do not have one or spending more time with their pets if they have one.

We find that the LIWC category on *anxiety* is more associated with users between 23 and 29; also, the LIWC category on *sadness* is more associated with users between 30 and 65.

### 3.2 Gender analysis

In the gender analysis, we observed that female and male users tended to publish posts on topic themes about issues with sleep and substance use (Tables 4 and 5). However, female users tended to publish posts on topic themes about expressing their emotions such as happiness/sadness/anger and feeling scared/mad/confused/upset/afraid/jealous (Table 4) while male users tend to post more on topics related to issues with trust/problems in relationships (Table 5). Some of these findings align with the findings from prior work; for example, in [41], it was demonstrated that, on social media, there are distinctions in the way female and male users self-disclose information or concerns pertaining to their health and well-being and that female users tend to express more emotions such as anxiety and sadness compared to male users. A new insight from this work that was not determined in prior work is that male users who express loneliness on Twitter tend to express issues with trust in relationships compared to female users.

We observed that the LIWC categories on *sadness* and *focusing on the future* were more associated with female users and the LIWC categories on *female references*, *health*, and *anxiety* were more associated with male users.

### 3.3 Gender and age group analysis

#### 3.3.1 Same gender but different age groups

As it relates to the gender and age group analysis—specifically, users who belong to the same gender but different age groups, we observed that male users who express loneliness and are: (a) between the ages of 18 and 22 tend to post on topic themes related to intimacy, express more negative feelings/emotions, and post about mental health concerns (Table 6) and the LIWC category on *sadness* was more associated with users in this age group, (b) between the ages of 23 and 29 tend to post on topics related to dating and expressing compliments/how one should be treated with respect (Table 7) and the LIWC categories on *anxiety* and *social processes* were more associated with users in this age group, (c) between the ages of 30 and 65 tend to post about their family members (Table 8) and the LIWC categories on *leisure* was more associated with users in this age group.

We observed that female users who express loneliness and are: (a) between the ages of 18 and 22 tended to post on topic themes about intimacy and they tend to express their feelings/emotions such as feeling scared/confused/afraid/jealous (Table 9) and tended to use more words from the LIWC categories on *risk* and *health*, (b) between the ages of 23 and 29 tend to post on topic themes related to dating (Table 10) and tend to use words from the LIWC categories on *anxiety* and *focusing on the future*, and (c) between 30 and 65 tended to post on topics about communicating with family members, how people treat/view/talk about others, and about their mistakes and regrets (Table 11) and tend to user more words from the LIWC category *Hear*. Prior work [41], showed that female users who express health and well-being concerns on social media tend to share information related to their family members; in this work, we find that female users between the ages of 30 and 65 who express loneliness on Twitter tended to post more on topics about communicating with their family members compared to female users between the ages of 18 and 22 and 23 and 29. These findings from the gender and age group analysis indicate that there are distinctions in the support needs/concerns expressed on Twitter posts by users who belong to the same gender but different age groups, hence, online interventions around loneliness should take this into consideration. For example, given that male users between the ages of 18 and 22 tend to post about negative emotions and mental health concerns and female users in this age group also tend to post on topics related to emotions such as feeling scared/confused/afraid/jealous, online loneliness interventions for users belonging to these age group and are either female or male, should provide mental health counseling services.

#### 3.3.2 Same age group but different genders

As it relates to the gender and age group analysis—specifically, users who belong to the same age group but different genders, we observed that female users who express loneliness on Twitter and are between the ages of 18 and 22 tended to post more on topics themes about issues with insomnia and tended to post about sports (Table 9) and use more words from the LIWC categories on *risk* and *feel* compared to male users in this age group (who express loneliness) who tend to post more on topics related to fake friends/family, being bored/irritated/tired/sleepy, about the looks of men/women, and getting drunk (Table 6) and use more words from the LIWC categories on *sadness* and *female references*. Female users between 23 and 29 tended to post about topics themes on mental health concerns (Table 10) and use more words from the LIWC categories on *sadness*, *feel*, and *focus future* compared to male users in this age group (who express loneliness on Twitter) who tended to post on topics about fake friends/family, being bored/irritated/tired/sleepy, about the looks of men/women, and getting drunk, and communicating with family members (Table 7) and tended to use more words from the LIWC categories on *female references*, *swearing*, *social processes*, and *health*. Female users between the ages of 30 and 65 tended to post on topics about communicating with family members, how people treat/view/talk about others, companionship, issues with trust in relationships, issues with insomnia, and their mistakes and regrets (Table 11) and tended to use more words from the LIWC categories on *feelings*, *negations*, *sadness*, and *negative emotion* compared to male users in this age group who tended to post on topics about feeling sick and tended to post about their family members as it relates to marriage (Table 8) and tended to use more words from the LIWC category on *leisure*. These findings show that there are clear distinctions in the support needs/concerns expressed in Twitter posts by users (who express loneliness) who belong to the same age group but different genders, therefore online loneliness interventions should be cognizant of these differences. For example, given that female users in the age group *30—65* tended to post on topic themes related to mistakes and regrets they have, online loneliness interventions may provide counseling services around this. Also, given that male users who express loneliness and belong to the age groups: *18—22* and *23—29* tend to post on topic themes related to drinking, online loneliness interventions can provide counseling around drinking and substance use to users in this group.

### 3.4 Suggestions for the design/implementation of online loneliness interventions

Prior work showed that Twitter posts can be mapped to the county level [42], hence, some loneliness interventions can be implemented both online and offline. Here, we suggest some online and offline loneliness interventions based on the findings from this work: (a) given that users in the age group of 18 and 22 and those between 23 and 29 tend to post on topic themes on sleep and substance use compared to users between 30 and 65, an online loneliness intervention could provide to users in this age group links related to tips on how to sleep well and advertisements and campaigns on how to quit/reduce substance use. Also, given that the county in which a user is posting on Twitter from can be determined [42], an online loneliness intervention can recommend sleep studies being conducted by credible research institutes in close proximity to where the user is located and in the case of substance use, the online loneliness intervention can suggest nearby substance use recovery facilities/substance use recovery counselors to these users (b) given that the county from which a user is publishing Twitter posts from can be determined and that users between the ages of 30 and 65 who express loneliness on Twitter tend to post more on topic themes related to pets, an online loneliness intervention can, for example, suggest to users in this age group (who express loneliness on Twitter) local pet clubs or pet shelters (that are close to the county from which they publish their Twitter posts) were they could meet with other individuals with shared interests in pets/interact with pets.

Online loneliness interventions have to be designed in such a way that user privacy is respected. Also, when designing and implementing an online loneliness intervention several factors need to be considered and addressed; for example, how can it be determined if an intervention is the right one for a user and if a user publishes posts related to self-harm, who should intervene?

Similar to prior work [41] that suggested that mental health interventions should be more gender aware and culture aware, the findings in this work indicate that online loneliness interventions need to be gender and age aware in order to provide adequate support to individuals who express loneliness on social media.

## 4 Limitations and future work

The study sample used for the analysis in this work comprises of social media users and is not representative of the population at large. Given that the inclusion criteria in this work is based on the number of tweets mentioning *“alone”* or *“lonely”* and users that have more than 50 twitter posts, we cannot extrapolate about those users who have fewer than 50 tweets or those that express loneliness in other ways other than using the words *“alone”* or *“lonely”*. In the future, we aim to analyze posts and comments from several online loneliness forums to gain insights as to the types of social support individuals seek (as it relates to loneliness) on these forums.

The Twitter posts used in this work were collected from users in a state (Pennsylvania) in the United States and may not be representative of all users who express loneliness either on social media or other online forums.

In this work, we conducted analysis on the following genders: female and male. In the future, we will conduct analysis to determine the differences in the entire gender spectrum.

In this work, we analyzed Twitter posts of users who expressed loneliness by mentioning the words *“alone”* or *“lonely”* in their Twitter posts. Prior work [33], indicated that the words “alone” and “lonely” are conceptually different; in the future, we will conduct analysis to determine if there are differences in the use of the words “alone” and “lonely” across age groups and genders.

With this work, it is our hope that more work will be done to provide online interventions around loneliness.

## 5 Conclusion

In this work, we used LDA and LIWC to show that there are differences in the use of language by female and male users who express loneliness on Twitter and the same applies to users in different age groups. We also determine that there are differences in the use of language by users who belong to different genders and age groups and express loneliness on Twitter. We observe that these differences in language use reflect the difference in support needs and concerns expressed by users in these different groups. Knowing these variations in language use is important for designing and providing online interventions to individuals who express loneliness on Twitter.
